# Abnormal Cardiac Repolarization in Thyroid Diseases: Results of an Observational Study

**DOI:** 10.3389/fcvm.2021.738517

**Published:** 2021-11-23

**Authors:** Assem Aweimer, Fabian Schiedat, Dominik Schöne, Gabi Landgrafe-Mende, Harilaos Bogossian, Andreas Mügge, Polykarpos C. Patsalis, Michael Gotzmann, Ibrahim Akin, Ibrahim El-Battrawy, Johannes W. Dietrich

**Affiliations:** ^1^Cardiology and Angiology Department, Medical Hospital II, Bergmannsheil University Hospitals, Ruhr University of Bochum, Bochum, Germany; ^2^Department of Cardiology, St. Mary's Hospital, University of Duisburg-Essen, Gelsenkirchen, Germany; ^3^Diabetes, Endocrinology and Metabolism Section, Department of Medicine I, St. Josef Hospital, Ruhr-University of Bochum, Bochum, Germany; ^4^Cardiology and Rhythmology Department, EvK Hospital Hagen-Haspe, Witten-Herdecke University, Witten, Germany; ^5^Department of Cardiology, University Hospital St. Josef Hospital, Ruhr University Bochum, Bochum, Germany; ^6^First Department of Medicine, Faculty of Medicine, University Medical Centre Mannheim, University of Heidelberg, Mannheim, Germany; ^7^Ruhr Centre of Rare Diseases, Ruhr University of Bochum, Bochum, Germany; ^8^Ruhr Centre of Rare Diseases, Witten-Herdecke University, Witten, Germany; ^9^Diabetes Centre Bochum/Hattingen, Blankenstein Hospital, Hattingen, Germany

**Keywords:** repolarization, T-peak-to-end interval, JT interval, thyroid hormones, thyroid disorder and heart

## Abstract

**Background:** The relationship between thyroid function and cardiac disease is complex. Both hypothyroidism and thyrotoxicosis can predispose to ventricular arrhythmia and other major adverse cardiovascular events (MACE), so that a U-shaped relationship between thyroid signaling and the incidence of MACE has been postulated. Moreover, recently published data suggest an association between thyroid hormone concentration and the risk of sudden cardiac death (SCD) even in euthyroid populations with high-normal FT4 levels. In this study, we investigated markers of repolarization in ECGs, as predictors of cardiovascular events, in patients with a spectrum of subclinical and overt thyroid dysfunction.

**Methods:** Resting ECGs of 100 subjects, 90 patients (LV-EF > 45%) with thyroid disease (60 overt hyperthyroid, 11 overt hypothyroid and 19 L-T4-treated and biochemically euthyroid patients after thyroidectomy or with autoimmune thyroiditis) and 10 healthy volunteers were analyzed for Tp-e interval. The Tp-e interval was measured manually and was correlated to serum concentrations of thyroid stimulating hormone (TSH), free triiodothyronine (FT3) and thyroxine (FT4).

**Results:** The Tp-e interval significantly correlated to log-transformed concentrations of TSH (Spearman's rho = 0.30, *p* < 0.01), FT4 (rho = −0.26, *p* < 0.05), and FT3 (rho = −0.23, *p* < 0.05) as well as log-transformed thyroid's secretory capacity (SPINA-GT, rho = −0.33, *p* < 0.01). Spearman's rho of correlations of JT interval to log-transformed TSH, FT4, FT3, and SPINA-GT were 0.51 (*p* < 1e−7), −0.45 (*p* < 1e−5), −0.55 (*p* < 1e−8), and −0.43 (*p* < 1e−4), respectively. In minimal multivariable regression models, markers of thyroid homeostasis correlated to heart rate, QT, Tp-e, and JT intervals. Group-wise evaluation in hypothyroid, euthyroid and hyperthyroid subjects revealed similar correlations in all three groups.

**Conclusion:** We observed significant inverse correlations of Tp-e and JT intervals with FT4 and FT3 over the whole spectrum of thyroid function. Our data suggest a possible mechanism of SCD in hypothyroid state by prolongation of repolarization. We do not observe a U-shaped relationship, so that the mechanism of SCD in patients with high FT4 or hyperthyroidism seems not to be driven by abnormalities in repolarization.

## Introduction

The relationship of thyroid disorders and cardiovascular diseases raises growing interest (1–3). Both, hypothyroid or hyperthyroid conditions may lead to increased cardiovascular morbidity and mortality (4). This well-accepted U-shaped relationship between thyroid function and cardiac disease is, however, still not fully clarified (5). As far as we know today, the mechanisms of hypo- or hyperthyroidism leading to cardiac diseases are diverse with effects on cardiac contractility, vasculature and cardiac electrophysiology (6, 7).

In a general population study an association between thyroid hormone levels, even within the respective normal range, and ECG changes has been described (8). Published data suggest an association between thyroid hormone concentrations and the risk of sudden cardiac death (SCD) even in euthyroid populations with high-normal FT4 concentration (9). Recently, similar relations between thyroid function and the incidence of other endpoints, including malignant arrhythmia and stress cardiomyopathy (Takotsubo syndrome), have been described (10, 11). However, the electrophysiological mechanisms underlying these observations are poorly understood up to now.

Repolarization abnormalities, especially prolonged repolarization, are assumed to be among the major risk factors for SCD (12, 13). Contradicting observations have been published concerning the effect of thyroid hormone levels especially on the QTc interval (14–18). Whereas, the effect of thyroid hormone disorders on the QTc interval has been extensively studied, very little is known on ventricular repolarization as measured by more specific repolarization markers including Tpeak-to T-end interval (Tp-e) or JT interval. The Tp-e interval has been established and recognized as a correlate of dispersion of ventricular repolarization (19). A prolonged Tp-e interval, and therefore a longer dispersion of ventricular repolarization, may be associated with ventricular tachyarrhythmias (20); similar findings were reported regarding the JT interval (13).

Interestingly, a prolonged Tp-e interval seems independently associated with SCD, even if the QTc is normal (21). Recently, one study showed a prolongation of Tp-e interval in patients with subclinical hypothyroidism (22). However, data on patients with overt thyroid disorders and hyperthyroidism have not been published up to now.

In this study, we investigated the Tp-e and JT intervals in ECGs of patients with a spectrum of subclinical and overt thyroid dysfunction and a euthyroid control group to assess pathological repolarization as a potential indicator of SCD.

## Methods

### Study Design and Population

This study included 90 patients of the NOMOTHETICOS cohort (23) with untreated and treated primary thyroid dysfunction and for comparison 10 healthy volunteers. In all subjects a full thyroid hormone profile and 12-lead ECG measurements were performed.

None of the subjects had a severely impaired left ventricular ejection fraction (LV-EF < 45%) or a left bundle branch block.

Additional inclusion criteria for patients with thyroid disease were

- Disconnected feedback control due to the following conditions at the time of recruitment:
  ° Overt primary hypothyroidism with TSH level being higher than 10 mIU/l and FT4 concentration below 7 pmol/l (5.4 ng/l) (group 1)
  ° Overt primary hyperthyroidism with TSH level below 0.1 mIU/l and FT4 concentration higher than 18 pmol/l (14 ng/l) (group 3)
  ° All other constellations, if the patient receives substitution therapy with more than 1.75 μg Levothyroxin per kg of body mass (group 2).
- System in equilibrium (requiring no or unchanged substitution dose over the previous 6 weeks before investigation).

Inclusion criterion for healthy volunteers (group 0) were normal thyroid function with ruled out thyroid disease via ultrasound and normal concentrations of antibodies to thyroid peroxidase (TPOAb), thyroglobulin (TgAb), and TSH receptors (TRAb).

Exclusion criteria for all subjects were pituitary or hypothalamic disease, resistance to thyroid hormone, Allan-Herndon-Dudley syndrome, severe illness possibly associated with non-thyroidal illness syndrome (TACITUS), medication influencing pituitary function and pregnancy.

All subjects delivered written informed consent. The study design was approved by the local institutional ethics committee of the Medical Faculty at the Ruhr University of Bochum under the file number 3718-10. The protocol of the NOMOTHETICOS study (UTN U1111-1122-3273) has been registered at ClinicalTrials.gov (ID NCT01145040) and in the German Clinical Trials Register (ID DRKS00003153).

### ECG Measurements

**Figure 1:**
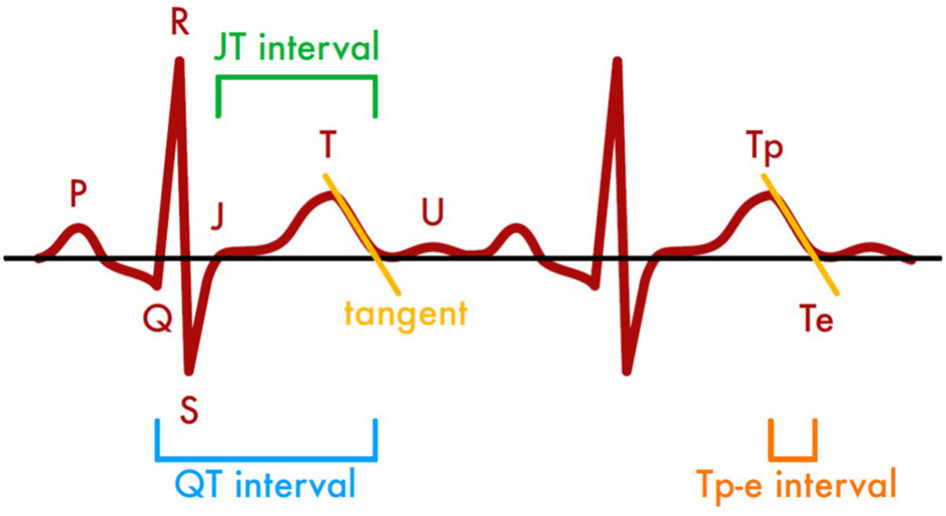
Methods of assessing time intervals in ECG recordings based on the tangent method.

All anonymized ECG recordings were distributed to three independent cardiologists blinded for laboratory results. The QT interval, defined as beginning of Q wave to the end of T wave, the JT interval, defined as the period from the J point (junction between the termination of the QRS complex and the beginning of the ST segment) to the end of the T wave, and Tp-e interval, defined as the distance of T wave peak to the end of T wave, were measured (Figure 1). The end of the T wave was determined by the tangent method (24). All measurements were performed manually and preferentially in lead V5. If a measurement in V5 was not possible the investigators were instructed to select alternatively lead V2–V4 or lead II (25).

### Laboratory Measurements

Serum concentrations of TSH, FT4, and FT3 were determined with a fully automated chemiluminescence-based system (DxI800, Beckman-Coulter, Krefeld, NRW, Germany). The intra-assay and inter-assay CVs for these analyses vary with concentrations but are <10% for the range of measurement. Thyroid tissue antibodies (TPOAb, TgAb, and TRAb) were measured with quantitative radioimmunoassays (anti-TPOn, anti-Tgn and TRAKhuman, ThermoFisher, BRAHMS division, Henningsdorf, BB, Germany).

To assess the relative contributions of the pituitary and thyroid gland to the variations in hormone concentrations, Jostel's TSH index (JTI), thyroid's secretory capacity (SPINA-GT), and the sum activity of peripheral deiodinases (SPINA-GD) were calculated from steady-state concentrations of TSH and FT4 and constants for plasma protein binding and kinetics, as recently recommended for thyroid trial design (23, 26).

### Statistical Analysis

Statistical analyses were performed with custom S scripts written for the environment R 3.6.3 on macOS. Depending on the class of analyzed data and possible direction of causality, distributions were compared with multivariable regression, 2-sample Student's *t*-test (Gaussian distributed variables), Wilcoxon's rank sum test (non-Gaussian variables) or chi-square test (counts). All four groups were compared with one-way ANOVA and *post-hoc* pairwise *t*-test. Alpha error correction for multiple testing was performed with the Benjamini-Hochberg procedure. Continuous variables were tested for normality with quantile-quantile (Q-Q) plots of z-transformed values.

To identify parameters that independently correlate to electrophysiological markers we conducted step-wise multivariable regression analysis based on variables that had a significant association to ECG markers in a univariable investigation. For this purpose, an initial maximal model including all possible predictors as suggested by univariable analysis was successively simplified by eliminating non-significant parameters to deliver a final minimal model containing significant predictors only. Thyroid volume and calculated parameters of thyroid homeostasis were not included in multivariable models in order to avoid multicollinearity. Before inclusion in a multivariable model TSH and thyroid hormone concentrations were logarithmically transformed to meet criteria of a normal distribution.

Where not otherwise specified, data are presented as mean value ± standard error of the mean (SEM) for normally distributed continuous data, as median (interquartile range) for non-normally distributed data or as absolute numbers (percentages) for count data. A *p*-value < 0.05 was considered statistically significant.

## Results

### Clinical Characteristics

One hundred subjects were included in the analysis, 90 patients with thyroid disease (60 overt hyperthyroid, 11 overt hypothyroid and 19 L-T4-treated and biochemically euthyroid patients after thyroidectomy or with autoimmune thyroiditis) and 10 healthy volunteers for comparison purposes (Table 1). The mean age of the subjects was 52.8 ± 1.60 years, and 69 patients were women.

**Table 1:** Basic characteristics of study population.

	**Group 0 (normal subjects)**	**Group 1 (hypothyroidism)**	**Group 2 (full-dose L-T4 substitution therapy)**	**Group 3 (thyrotoxicosis)**
N	10	11	19	60
Age (years)	56.3 ± 4.9	56.7 ± 4.6	45.1 ± 3.0	54.0 ± 2.1
Female (%)	6 (60%)	6 (54%)	17 (89%)	40 (67%)
BMI (kg/m^2^)	27.5 ± 1.0	32.7 ± 3.4^††^	25.1 ± 1.7**	25.0 ± 0.7**
Atrial fibrillation (%)	0 (0%)	1 (9%)	0 (0%)	10 (17%)
**Thyroid disease**				
Acute or subacute thyroiditis	0	0	0	8
Amiodarone-induced thyroid disease	0	1	0	7
Toxic adenoma	0	0	1	5
Hashimoto's disease	0	0	1	1
Ord's disease	0	5	0	0
Graves' disease	0	2	2	16
Graves' disease with Hashimoto component	0	1	3	20
Schmidt-Carpenter's syndrome	0	0	1	0
Marine-Lenhart syndrome	0	0	0	3
Post-surgical or radiogenic hypothyroidism	0	2	11	0
Sodium (135–145 mmol/L)	138.8 ± 0.6	139.9 ± 1.0	139.6 ± 0.9	138.8 ± 0.4
Potassium (3.5–5 mmol/L)	4.1 ± 0.2	4.1 ± 0.1	4.1 ± 0.1	4.1 ± 0.1
Calcium (2.2–2.6 mmol/L)	2.3 ± 0.0	2.2 ± 0.1	2.2 ± 0.1^†^	2.4 ± 0.0
Creatinine (71–124 μmol/L)	75.1 (35.4)^†^	97.2 (35.4)^†††^	61.9 (8.8)*	61.9 (26.5)***
HbA1c (4–6%)	5.8 ± 0.3	6.4 ± 0.5	5.6 ± 0.3	5.6 ± 0.1
Beta1-selective beta-blocker use	3 (30%)	4 (36%)	5 (26%)	27 (45%)
Unselective beta-blocker use	0 (0%)^†††^	0 (0%)^†††^	0 (0%)^†††^	21 (35%)***^‡‡‡^
Cardiac glycoside use	0 (0%)	1 (9%)	0 (0%)	2 (3%)
Amiodarone use	0 (0%)	1 (9%)	0 (0%)	7 (12%)
Carbamazepine use	0 (0%)	0 (0%)	1 (5%)	0 (0%)
Methylphenidate use	0 (0%)	0 (0%)	1 (5%)	0 (0%)
Antipsychotic drug use	0 (0%)	1 (9%)	1 (5%)	2 (3%)
Selective serotonin reuptake inhibitor use	0 (0%)	0 (0%)	1 (5%)	2 (3%)
Tricyclic antidepressant used	2 (20%)	0 (0%)	1 (5%)	2 (3%)
Antihistamine use	0 (0%)	1 (9%)	0 (0%)	1 (2%)
Thyroid volume (mL)	11.0 ± 0.6	12.3 ± 3.4^††^	8.4 ± 3.2^†††^	27.9 ± 2.1**
Levothyroxine dosage (μg/day)	0 ± 0	52.3 ± 23.0	88.2 ± 22.7^†††‡‡‡^	8.3 ± 5.3

Corrected p < 0.05 (*), < 0.01 (**), < 0.001 (***) compared to group 1 (hypothyroidism). Corrected p < 0.05 (^†^), < 0.01 (^††^), < 0.001 (^†††^) compared to group 3. Corrected p < 0.001 (^‡‡‡^) compared to group 0. See Supplementary Tables 1–20 for detailed p-values.

### Phenotype of Thyroid Homeostasis

As shown in Table 2, TSH and thyroid hormones of subjects in group 0 were in the respective reference ranges. This also applies to calculated parameters for thyroid output (SPINA-GT), total deiodinase activity (SPINA-GD) and Jostel's TSH index and, with the exception of a slightly higher mean TSH concentration, to all markers in group 2. Deiodinase activity was elevated in group 1, representing an activated TSH-T3 feedforward control in the setting of elevated TSH concentrations (Table 2).

**Table 2:** Measured and calculated parameters of thyroid homeostasis in the four groups.

**Parameter (reference range)**	**Group 0 (normal subjects)**	**Group 1 (hypothyroidism)**	**Group 2 (full-dose L-T4 substitution therapy)**	**Group 3 (thyrotoxicosis)**
TSH (0.35–3.5 mIU/L)	1.47 (0.91)***^††††^	76.33 (92.48)^††††‡‡‡^	1.41 (3.33)****^††††^	0.03 (0.04)****^‡‡‡‡^
FT4 (8–18 pmol/L)	12.2 (3.2)***^††††^	3.9 (2.3)****^††††‡‡‡^	12.9 (3.9)****^††††^	45.0 (22.5)****^‡‡‡‡^
FT3 (3.5–6.3 pmol/l)	4.9 (0.6)***^††††^	3.0 (0.8)^††††‡‡‡^	4.7 (1.0)****^††††^	16.1 (13.6)****^‡‡‡‡^
SPINA-GT (1.4–8.7 pmol/s)	2.74 (1.24)***^††††^	0.31 (0.18)^††††‡‡‡^	N/A	307.27 (940.48)****^‡‡‡‡^
SPINA-GD (20–60 nmol/s)	34.7 (7.1)****	62.9 (50.6)^††††‡‡‡^	35.4 (15.9)****^††††^	30.2 (16.2)****
JTI (1.3–4.1)	2.0 ± 0.2*	4.9 ± 0.2^†^	1.6 ± 0.5**	2.7 ± 0.4*

Corrected p < 0.05 (*), < 0.01 (**), < 0.001 (***), < 1e−4 (****) compared to group 1. Corrected p < 0.05 (^†^), < 0.01 (^††^), < 0.001 (^†††^), < 1e−4 (^††††^) compared to group 3. Corrected p < 0.001 (), < 1e−4 () compared to group 0.

**Figure 2:**
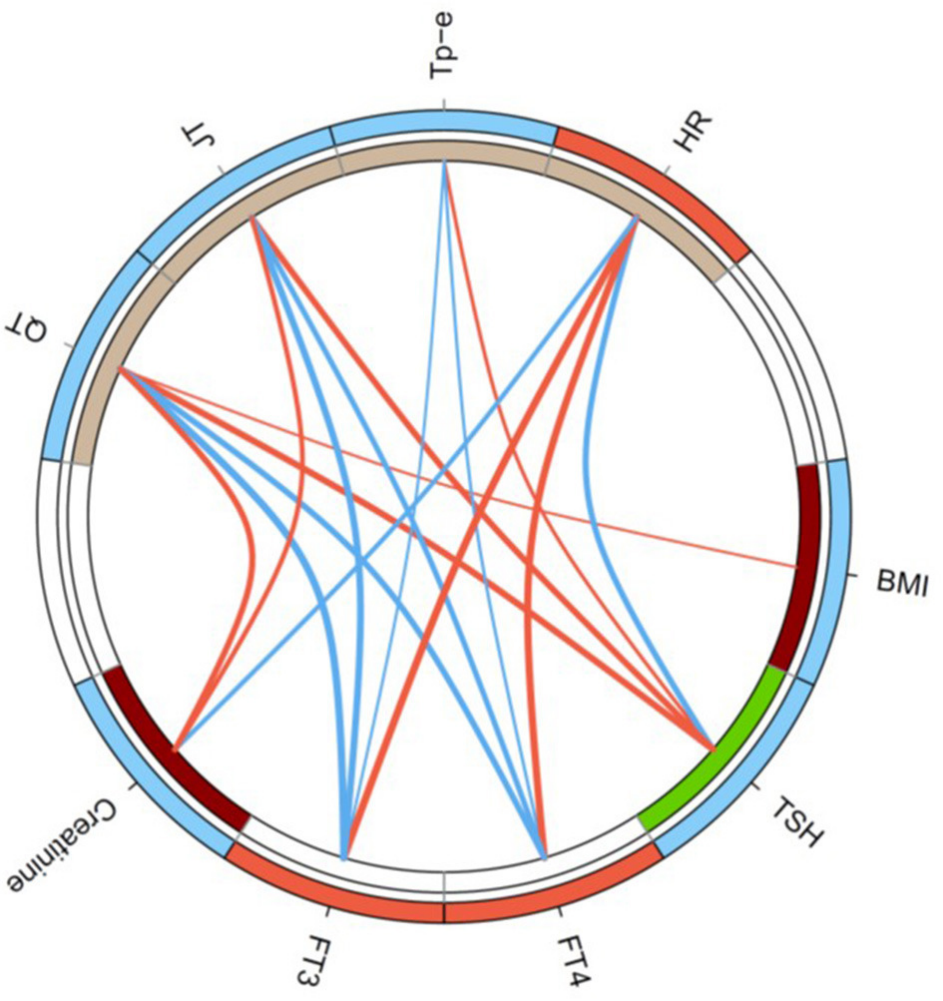
Circular map of the univariable correlation network of ECG markers to clinical and endocrine parameters. Shown are significant correlations (*p* < 0.05) only, and line thickness indicates the strength of negative (blue) or positive (red) correlation. Colors of circle segments indicate increased (red) or decreased values (blue), respectively, in thyrotoxicosis compared to hypothyroidism (outer ring) and higher (dark red) or lower values (green) in atrial fibrillation (inner ring).

**Figure 3:**
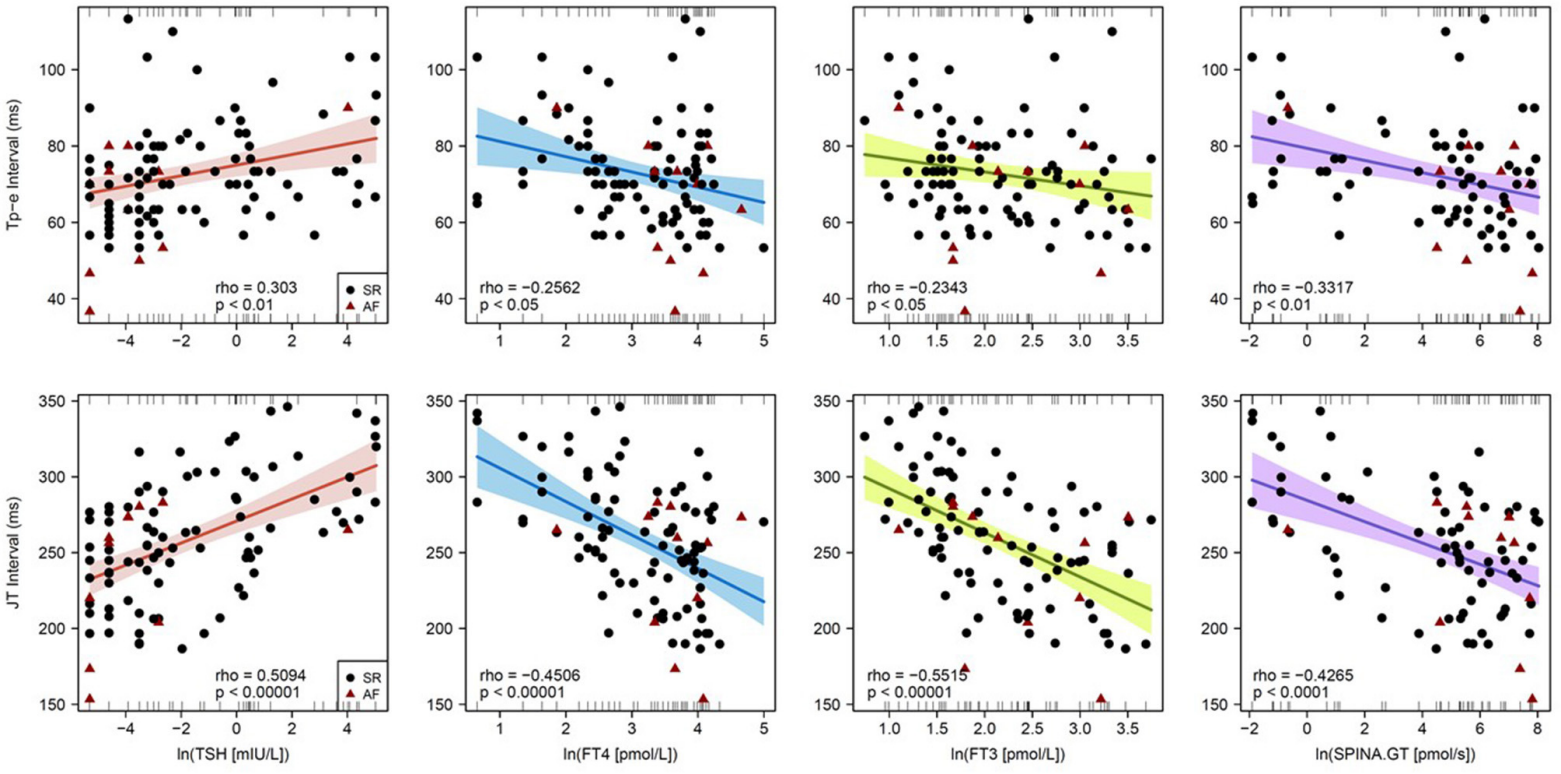
Correlations of Tp-e interval **(top)** and JT interval **(bottom)** with endocrine parameters of thyroid homeostasis. Shown are single data points, linear models from Pearson regression with 95% confidence intervals and models of Spearman correlation (in the bottom of each panel).

In univariable regression analysis, the correlation of heart rate to clinical and endocrine parameters demonstrated an inverse pattern to that of repolarization markers (Figure 2). In detail, the Tp-e interval significantly correlated to log-transformed concentrations of TSH (Spearman's rho = 0.30, *p* < 0.01), FT4 (rho = −0.26, *p* < 0.05), and FT3 (rho = −0.23, *p* < 0.05) as well as log-transformed thyroid's secretory capacity (SPINA-GT, rho = −0.33, *p* < 0.01). Spearman's rho of correlations of JT interval to log-transformed TSH, FT4, FT3, and SPINA-GT were 0.51 (*p* < 1e−7), −0.45 (*p* < 1e−5), −0.55 (*p* < 1e−8), and −0.43 (*p* < 1e−4), respectively (Figure 3). Group-wise evaluation in hypothyroid, euthyroid and hyperthyroid subjects revealed similar correlations in all three groups.

Consistently with the results of group-wise hormone statistics (Table 2) and regression analysis the heart rate and markers of repolarization were significantly different between the four groups (Table 3). The P wave duration did not differ among the groups. In minimal models of multivariable analysis markers of thyroid homeostasis remained predictors of electrophysiological parameters after adjustment for medication, and they were the only predictors of Tp-e and JT intervals after step-wise reduction (Table 4).

**Table 3:** Heart rhythm characteristics in the four groups.

	**Group 0 (normal subjects)**	**Group 1 (hypothyroidism)**	**Group 2 (full-dose L-T4 substitution therapy)**	**Group 3 (thyrotoxicosis)**
Heart rate (min^−1^)	75 ± 5^†††^	71 ± 6^††††^	77 ± 3^††††^	100 ± 3****
QT interval (ms)	362 ± 12*^†^	399 ± 9^††††^	368 ± 8*^††^	330 ± 5****
Tp-e interval (ms)	73 ± 3	83 ± 4^†^	73 ± 3	70 ± 2*
JT interval (ms)	280 ± 13^††^	297 ± 9^††††^	278 ± 8^†††^	241 ± 5****
P wave duration (ms)	94 ± 4	96 ± 4	92 ± 3	93 ± 2

Corrected p < 0.05 (*), < 1e4 (****) compared to group 1. Corrected p < 0.05 (^†^), < 0.01 (^††^), < 0.001 (^†††^), < 1e−4 (^††††^) compared to group 3.

**Table 4:** Minimal models of multivariable regression.

	**B**	**SE**	**Wald**	**d.f**.	***p***	**OR (95% CI)**	**VIF**
**Heart rate (min** ^**−1**^ **)**							
FT4 (pmol/L)^*^	12.92	2.06	39.2	1	1.1e−8	4.1e5 (7143–2.3e7)	1.02
Selective	8.25	3.91	4.4	1	0.04	3832.8 (1.8–8.2e6)	1.02
beta-blocker							
**QT interval (ms)**							
TSH (mIU/L)^*^	4.96	1.82	7.5	1	0.008	142.5 (4.1–5004.9)	2.42
FT3 (pmol/L)^*^	−18.97	7.29	6.8	1	0.01	5.8e−9 (3.6e−15–9.3e−3)	2.41
Amiodarone	34.88	13.16	7.0	1	0.009	1.4e15 (8900.1–2.2e26)	1.03
**Tp-e interval (ms)**							
TSH (mIU/L)^*^	1.39	0.43	10.2	1	0.002	4.0 (1.7–9.4)	N/A
**JT interval (ms)**							
TSH (mIU/L)^*^	4.18	1.73	5.8	1	0.02	65.4 (2.2–1953)	2.36
FT3 (pmol/L)^*^	−16.32	6.98	5.1	1	0.02	8.1e−8 (9.4e−14–0.07)	2.36

*Variables were chosen by a priori selection and step-wise model simplification as described in the Methods section. Initial maximal models included BMI, medication (selective and unselective beta blockers, amiodarone, antihistamines) and serum concentrations of TSH, FT4, FT3, calcium, and creatinine. B, Beta coefficient; SE, standard error; Wald, Chi-squared from Wald's test; d.f., degrees of freedom; OR, odds ratio; VIF, variance inflation factor*.

* *Logarithmically transformed to account for asymmetrical distribution*.

## Discussion

In this study we investigated a potential relationship between thyroid function and several markers of cardiac repolarization. The data reveal a robust association of heart rate and several time constants in the resting ECG, especially of the JT interval, to thyroid function. This connection is stronger than that to other physiological predictors of heart rhythm including BMI, renal function and serum electrolytes. With respect to heart rate and QT interval, biomarkers of thyroid homeostasis remained significant predictors of cardiac electrophysiology in minimal multivariable regression models adjusting for antiarrhythmic medication including selective betablockers and amiodarone. Step-wise model simplification arrived at the elimination of all predictors except TSH and/or FT3 for Tp-e and JT intervals, so that these ECG constants are explained by thyroid function only.

Subjects in group 2 were slightly younger than in the other groups. The difference wasn't significant, however, nor did the age correlate to TSH concentration, thyroid hormone concentration, heart rate, TPE interval or JT interval. We therefore don't expect the results to be distorted by age.

Unlike previous studies our observations extend to overt thyroid abnormalities, too. Our findings indicate a positive correlation of Tp-e and JT intervals to TSH concentration and an inverse correlation to FT4 and FT3 serum concentrations as well as to thyroid's secretory capacity (SPINA-GT).

The correlations on the level of current hormone concentrations are also reflected by consistent associations to diagnostic groups. The Tp-e and JT intervals were elevated in the hypothyroid and reduced in the thyrotoxic group. This also applied to the QT interval, whereas the heart rate showed an inverse association to the functional categories. Heart rate and time intervals were identical in euthyroid subjects (group 0) and patients receiving full-dose substitution therapy with levothyroxine (group 2), rendering them biochemically euthyroid. This observation indicates that the observed effects on ECG parameters are causally mediated by thyroid hormone concentration and not by accompanying effects of thyroid function, including nerval damage or impaired calcium homeostasis in the postoperative state, or autoimmunity.

An overt hypothyroid status results in a prolongation of cardiac repolarization. Overt hypothyroidism is accompanied by an excess of mortality of up to 34% in 5 years as long as the underlying thyroid hormone disorder is untreated or undertreated (27). Whereas, the reason for increased mortality in affected patients was discussed to be caused by comorbidities and frailty (27, 28) a large register-based study observed the mortality to be increased in subjects without comorbidities, too (29). The well-documented effects of thyroid hormones on myocardial physiology may explain the close association of cardiovascular morbidity to hypothyroidism. Overt hypothyroidism is able to induce heart failure via many effects including bradycardia, impaired systolic function, impaired left ventricular diastolic filling or diastolic hypertension (6). Although thyroid hormones have a strong impact on cardiac electrophysiology, the specific effects of overt hypothyroidism on novel repolarization markers have not been extensively studied up to now. Our observations demonstrate that the hypothyroid group, in addition to a decreased heart rate as expected, is hallmarked by significantly prolonged Tp-e and JT intervals, which are established markers for increased mortality risk. Therefore, major adverse cardiovascular endpoints in patients with overt hypothyroidism might as well result from arrhythmic events driven by repolarization abnormalities.

Previous studies arrived at conflicting results regarding the association of cardiac repolarization with thyroid function in the range of high T3 or T4 concentrations. Some reports described prolonged time constants of repolarization in thyrotoxicosis (14, 16, 30), whereas others reported an inverse association of repolarization intervals with thyroid hormones (15, 17). Our results, based on the whole range of thyroid function, including hypothyroid, euthyroid and hyperthyroid subjects, suggest a uniform inverse correlation of repolarization constants, especially the Tp-e and JT intervals, to thyroid hormones.

Cardiac repolarization or ventricular dispersion results from an interplay of ionic potassium currents, which are influenced by beta-adrenergic stimulation, depending particularly on the expression of beta-adrenoreceptors (31, 32). Kang et al. demonstrated potassium channels in human left ventricular tissue to be modulated by beta-adrenoceptors. Beta-adrenergic stimulation led to increased transmural dispersion of repolarization, resulting in elevated arrhythmogenic risk (31). Likewise, imbalances of sympathetic and vagal tone in hyperthyroidism and TSH-suppressive therapy were promotive for increased QT dispersion (33). Interestingly, increased dispersion of the QT interval was also observed in hypothyroidism (34), Akin et al. suggesting a U-shaped relationship between thyroid hormones and certain markers of cardiac electrophysiology.

Of note, thyroid hormones are able to modulate the susceptibility of the heart for catecholamines by stimulating the expression of beta-adrenoceptors in cardiomyocytes, with subsequent positive inotropic and chronotropic effects (35–38). Conversely, intracellular cAMP formed by activated beta-adrenoceptors is able to stimulate the expression of a number of genes including that of type 2 deiodinase (DIO2), which converts T4–T3 and 3,5-T2, i.e., to more active thyroid hormones (39). The resulting latch-like behavior of cardiomyocytes leads to bifurcation, i.e., a radically different response to catecholamines in situations of even slightly elevated thyroid hormone concentration.

Bosch et al. previously described a delay of repolarization in hypothyroidism to be mediated by decreasing potassium channel currents in the ventricular tissue of guinea pig hearts (40). Conversely, thyrotoxicosis is expected to trigger shortened cardiac repolarization. This would be in accordance with our results and prior studies (15, 17, 22).

In addition to the well-recognized thyroid hormones FT4 or FT3 even TSH can affect cardiac repolarization via TSH-receptors (TSHR) on cardiomyocytes by modulating potassium and calcium channel currents (41). Additionally, cAMP formed by TSHR activation may contribute to local hyperdeiodination and activation of the above-mentioned latch-like response.

Therefore, the distinct effects of thyrotropin and thyroid hormones on ventricular dispersion and its interaction with ionic currents via modulation of beta-adrenoceptors seem to be part of a complex scenario. Since both elevated iodothyronine and TSH concentrations are able to contribute to bifurcation, one might assume a U-shaped relationship between thyroid function and certain electrophysiological constants (5, 8). In our observations covering the whole range of thyroid function we didn't obtain, however, any hint for a U-shaped relationship. The increased risk of SCD in patients with high-normal FT4 concentration or thyrotoxicosis seems to be driven by mechanisms different from abnormalities in repolarization. This conclusion is also underscored by the fact that we saw in the thyrotoxic group only a moderate correlation of FT3 concentration to heart rate and the QT interval, but no correlations to other repolarization markers nor any correlations for other functional thyroid parameters (Supplementary Table 21).

Limitations of our approach are in the comparably small sample size and in the fact that subjects were recruited based on routine investigations, so that we were unable to determine non-classical thyroid hormones including 3,5-diiodothyronine (3,5-T2), thyronamines and iodothyroacetates, which may have a strong impact on cardiovascular physiology (42–44). Furthermore, our ECGs were not obtained digitally so that automatic measurements with a higher accuracy than the manual method could not be performed.

Strengths include multivariable modeling including medication, calcium and creatinine concentration, the evaluation of previously under-recognized biomarkers of cardiac electrophysiology and of calculated structure parameters of thyroid homeostasis, the determination of biologically more active free thyroid hormones and the inclusion of subjects from a broad range of thyroid function.

Motivated by previously observed but still poorly understood associations of SCD to within-reference range variations of thyroid hormone concentrations (3, 9–11), our research aimed at unveiling possible relationships between cardiac repolarization and derailed thyroid function. Our observations suggest that pathological repolarization might contribute to increased mortality in hypothyroidism. In thyrotoxicosis, however, the pathophysiology of major adverse cardiovascular end points seems to be mediated by mechanisms beyond repolarization.

## Data Availability Statement

The raw data supporting the conclusions of this article will be made available by the authors, without undue reservation.

## Ethics Statement

The studies involving human participants were reviewed and approved by the local Institutional Ethics Committee of the Medical Faculty at the Ruhr University of Bochum under the file number 3718-10.

## Author Contributions

AA, JD, AM, IA, and IE-B made substantial contributions to the study conception and design and to the drafting and critical revision of the manuscript for important intellectual content. AA and JD performed the statistical analysis. FS, PP, DS, GL-M, HB, and MG organized the database. All authors contributed to manuscript revision, read, and approved the submitted version.

## Funding

We acknowledge support by the Open Access Publication Funds of the Ruhr-Universität Bochum.

## Conflict of Interest

The authors declare that the research was conducted in the absence of any commercial or financial relationships that could be construed as a potential conflict of interest.

## Publisher's Note

All claims expressed in this article are solely those of the authors and do not necessarily represent those of their affiliated organizations, or those of the publisher, the editors and the reviewers. Any product that may be evaluated in this article, or claim that may be made by its manufacturer, is not guaranteed or endorsed by the publisher.
