# Supplementary material for: Abnormal Cardiac Repolarization in Thyroid Diseases: Results of an Observational Study
Source: Front Cardiovasc Med. 2021 Nov 23;8:738517. doi: 10.3389/fcvm.2021.738517 (PMC8649843; doi:10.3389/fcvm.2021.738517)
Supplement: Supplementary file 1 [file Data_Sheet_1.docx]

**Supplementary tables accompanying the publication "** **Abnormal cardiac repolarization in thyroid diseases: results of an observational study"**

*Assem Aweimer*, Fabian Schiedat, Dominik Schöne, Gabi Landgrafe-Mende, Harilaos Bogossian, Andreas Mügge, Polykarpos C. Patsalis, Michael Gotzmann, Ibrahim Akin, Ibrahim El-Battrawy, Johannes W. Dietrich*

*** Correspondence:** Corresponding Author: assem.aweimer@rub.de

**Supplementary table 1:** Pairwise *p* for age and group (see table 1 for basic statistical moments). Group 0: normal subjects; group 1: hypothyroidism; group 2: full-dose L-T4 substitution therapy; group 3: thyrotoxicosis).

|  | Group 0 | Group 1 | Group 2 |
| --- | --- | --- | --- |
| Group 1 | 0.95 | – | 0.14 |
| Group 2 | 0.14 | 0.14 | – |
| Group 3 | 0.81 | 0.81 | 0.14 |

**Supplementary table 2:** Pairwise *p* for body mass index and group.

|  | Group 0 | Group 1 | Group 2 |
| --- | --- | --- | --- |
| Group 1 | 0.1235 | – | **0.0091** |
| Group 2 | 0.3852 | **0.0091** | – |
| Group 3 | 0.3491 | **0.0038** | 0.9632 |

**Supplementary table 3:** Pairwise *p* for sodium concentration and group.

|  | Group 0 | Group 1 | Group 2 |
| --- | --- | --- | --- |
| Group 1 | 0.83 | – | 0.96 |
| Group 2 | 0.83 | 0.96 | – |
| Group 3 | 0.99 | 0.83 | 0.83 |

**Supplementary table 4:** Pairwise *p* for potassium concentration and group.

|  | Group 0 | Group 1 | Group 2 |
| --- | --- | --- | --- |
| Group 1 | 0.95 | – | 0.95 |
| Group 2 | 0.95 | 0.95 | – |
| Group 3 | 0.95 | 0.95 | 0.95 |

**Supplementary table 5:** Pairwise *p* for calcium concentration and group.

|  | Group 0 | Group 1 | Group 2 |
| --- | --- | --- | --- |
| Group 1 | 0.697 | – | 0.740 |
| Group 2 | 0.609 | 0.740 | – |
| Group 3 | 0.609 | 0.075 | **0.015** |

**Supplementary table 6:** Pairwise *p* for creatinine concentration and group.

|  | Group 0 | Group 1 | Group 2 |
| --- | --- | --- | --- |
| Group 1 | 0.21871 | – | **0.02434** |
| Group 2 | 0.45499 | **0.02434** | – |
| Group 3 | 0.19959 | **0.00033** | 0.21871 |

**Supplementary table 7:** Pairwise *p* for HbA1c fraction and group.

|  | Group 0 | Group 1 | Group 2 |
| --- | --- | --- | --- |
| Group 1 | 0.153 | – | 0.153 |
| Group 2 | 0.897 | 0.153 | – |
| Group 3 | 0.897 | **0.027** | 0.987 |

**Supplementary table 8:** Pairwise *p* for thyroid volume and group.

|  | Group 0 | Group 1 | Group 2 |
| --- | --- | --- | --- |
| Group 1 | 0.8928 | – | 0.7296 |
| Group 2 | 0.8928 | 0.7296 | – |
| Group 3 | 0.1171 | **0.0061** | **2.2e–5** |

**Supplementary table 9:** Pairwise *p* for levothyroxine dosage and group.

|  | Group 0 | Group 1 | Group 2 |
| --- | --- | --- | --- |
| Group 1 | 0.0681 | – | 0.1342 |
| Group 2 | **0.0007** | 0.1342 | – |
| Group 3 | 0.6802 | 0.0509 | **8.7e–6** |

**Supplementary table 10:** Pairwise *p* for TSH concentration and group.

|  | Group 0 | Group 1 | Group 2 |
| --- | --- | --- | --- |
| Group 1 | **0.00015** | – | **1.4e–5** |
| Group 2 | 0.63568 | **1.4e–5** | – |
| Group 3 | **1.3e–6** | **4.2e–7** | **9.8e–8** |

**Supplementary table 11:** Pairwise *p* for FT4 concentration and group.

|  | Group 0 | Group 1 | Group 2 |
| --- | --- | --- | --- |
| Group 1 | **0.00013** | – | **1.0e–5** |
| Group 2 | 1.00000 | **1.0e–5** | – |
| Group 3 | **9.8e–7** | **4.8e–7** | **3.8e–10** |

**Supplementary table 12:** Pairwise *p* for FT3 concentration and group.

|  | Group 0 | Group 1 | Group 2 |
| --- | --- | --- | --- |
| Group 1 | **0.00015** | – | **5.2e–5** |
| Group 2 | 0.58093 | **5.2e–5** | – |
| Group 3 | **1.3e–6** | **4.9e–7** | **8.9e–10** |

**Supplementary table 13:** Pairwise *p* for SPINA-GT and group. *not calculated in subjects on L-T4.

|  | Group 0 | Group 1 | Group 2 |
| --- | --- | --- | --- |
| Group 1 | **0.00012** | – | –* |
| Group 2 | –* | –* | –* |
| Group 3 | **7.5e–7** | **4.9e–7** | –* |

**Supplementary table 14:** Pairwise *p* for SPINA-GD and group.

|  | Group 0 | Group 1 | Group 2 |
| --- | --- | --- | --- |
| Group 1 | **0.00098** | – | **8.9e–10** |
| Group 2 | 0.8007 | **0.00016** | – |
| Group 3 | 0.11795 | **4.8e–6** | 0.11795 |

**Supplementary table 15:** Pairwise *p* for JTI and group.

|  | Group 0 | Group 1 | Group 2 |
| --- | --- | --- | --- |
| Group 1 | **0.0144** | – | **0.0032** |
| Group 2 | 0.6844 | **0.0032** | – |
| Group 3 | 0.4747 | **0.0144** | 0.1351 |

**Supplementary table 16:** Pairwise *p* for heart rate and group.

|  | Group 0 | Group 1 | Group 2 |
| --- | --- | --- | --- |
| Group 1 | 0.78799 | – | 0.71171 |
| Group 2 | 0.84376 | 0.71171 | – |
| Group 3 | **0.00059** | **5.5e–5** | **5.5e–5** |

**Supplementary table 17:** Pairwise *p* for mean Tp-e interval and group.

|  | Group 0 | Group 1 | Group 2 |
| --- | --- | --- | --- |
| Group 1 | 0.158 | – | 0.151 |
| Group 2 | 0.949 | 0.151 | – |
| Group 3 | 0.676 | **0.023** | 0.598 |

**Supplementary table 18:** Pairwise *p* for mean QT interval and group.

|  | Group 0 | Group 1 | Group 2 |
| --- | --- | --- | --- |
| Group 1 | **0.0402** | – | **0.0402** |
| Group 2 | 0.6990 | **0.0402** | – |
| Group 3 | **0.0380** | **2.5e–6** | **0.0012** |

**Supplementary table 19:** Pairwise *p* for mean JT interval and group.

|  | Group 0 | Group 1 | Group 2 |
| --- | --- | --- | --- |
| Group 1 | 0.33214 | – | 0.22944 |
| Group 2 | 0.86244 | 0.22944 | – |
| Group 3 | **0.00386** | **3.8e–5** | **0.00057** |

**Supplementary table 20:** Pairwise *p* for mean P wave duration and group.

|  | Group 0 | Group 1 | Group 2 |
| --- | --- | --- | --- |
| Group 1 | 0.8 | – | 0.8 |
| Group 2 | 0.8 | 0.8 | – |
| Group 3 | 0.8 | 0.8 | 0.8 |

**Supplementary table 21:** Group-wise correlations of TSH, thyroid hormones and SPINA-GT with TP-e and JT interval. Shown are *rho* and *p* values from Spearman’s rank correlation.

|  | TSH | | FT4 | | FT3 | | SPINA-GT | |
| --- | --- | --- | --- | --- | --- | --- | --- | --- |
|  | *rho* | *p* | *rho* | *p* | *rho* | *p* | *rho* | *p* |
| Hypothyroidism (Group 1, n=11) |  |  |  |  |  |  |  |  |
| Heart rate (min-1) | –0.58 | 0.06 | **0.64** | **0.03** | 0.32 | 0.34 | **0.69** | **0.02** |
| QT interval (ms) | 0.28 | 0.41 | –0.03 | 0.93 | –0.15 | 0.67 | –0.10 | 0.78 |
| Tp-e interval (ms) | 0.02 | 0.95 | 0.46 | 0.15 | 0.04 | 0.91 | 0.39 | 0.23 |
| JT interval (ms) | **0.67** | **0.02** | **–0.60** | **0.05** | –0.20 | 0.56 | **–0.62** | **0.04** |
| P wave duration (ms) | –0.48 | 0.16 | **0.65** | **0.05** | 0.25 | 0.48 | 0.61 | 0.06 |
| Euthyroidism (Groups 0 and 2, n=29) |  |  |  |  |  |  |  |  |
| Heart rate (min-1) | –0.16 | 0.41 | 0.25 | 0.18 | 0.17 | 0.38 | **0.84** | **0.002** |
| QT interval (ms) | 0.27 | 0.16 | –0.15 | 0.44 | –0.33 | 0.08 | –0.49 | 0.15 |
| Tp-e interval (ms) | –0.05 | 0.81 | **–0.52** | **0.004** | –0.11 | 0.56 | –0.23 | 0.53 |
| JT interval (ms) | 0.24 | 0.22 | –0.03 | 0.89 | –0.28 | 0.14 | –0.28 | 0.43 |
| P wave duration (ms) | 0.02 | 0.92 | –0.19 | 0.31 | –0.12 | 0.55 | –0.31 | 0.38 |
| Thyrotoxicosis (Group 3, n=60) |  |  |  |  |  |  |  |  |
| Heart rate (min-1) | 0.03 | 0.84 | 0.19 | 0.15 | **0.27** | **0.03** | 0.01 | 0.93 |
| QT interval (ms) | 0.14 | 0.28 | –0.22 | 0.57 | **–0.30** | **0.02** | –0.12 | 0.35 |
| Tp-e interval (ms) | 0.22 | 0.09 | –0.07 | 0.76 | –0.05 | 0.69 | –0.22 | 0.08 |
| JT interval (ms) | 0.09 | 0.48 | –0.04 | 0.96 | –0.24 | 0.06 | –0.06 | 0.64 |
| P wave duration (ms) | –0.02 | 0.91 | –0.01 | 0.13 | –0.13 | 0.36 | 0.01 | 0.96 |
| All subjects (Groups 0–3, n=100) |  |  |  |  |  |  |  |  |
| Heart rate (min-1) | **–0.49** | **2.2e–7** | **0.58** | **1.9e–10** | **0.61** | **2.4e–11** | **0.43** | **7.6e–5** |
| QT interval (ms) | **0.54** | **6.6e–9** | **–0.5** | **1.4e–7** | **–0.60** | **5.4e–11** | **–0.47** | **1.2e–5** |
| Tp-e interval (ms) | **0.30** | **0.002** | **–0.26** | **0.01** | **–0.23** | **0.02** | **–0.33** | **0.003** |
| JT interval (ms) | **0.51** | **6.3e–8** | **–0.45** | **2.5e–6** | **–0.55** | **2.7e–9** | **–0.43** | **7.2e–5** |
| P wave duration (ms) | 0.04 | 0.73 | –0.13 | 0.23 | –0.09 | 0.40 | –0.06 | 0.60 |
